# Trials, tribulations, and formulations in access to new TB drugs for children: learning from rifapentine

**DOI:** 10.5588/ijtldopen.26.0263

**Published:** 2026-10-12

**Authors:** A.C. Hesseling, N. Salazar-Austin, M. Louey, L. van der Laan, M. Gombe, T. Masini, M. Penazzato, I. Courtney, A. Garcia-Prats, D. Tarlton, M. Frick

**Affiliations:** 1 Desmond Tutu TB Centre, Department of Paediatrics and Child Health, Faculty of Medicine and Health Sciences, Stellenbosch University, Stellenbosch, South Africa;; 2 Department of Pediatrics and Johns Hopkins Bloomberg School of Public Health, Department of International Health, Johns Hopkins University School of Medicine, Baltimore, MD, USA;; 3 Clinton Health Access Initiative, New York, NY, USA;; 4 The Aurum Institute, Johannesburg, South Africa;; 5 Independent Consultant, Lucca, Italy;; 6 Global Accelerator for Paediatric Formulations Network, World Health Organization, Geneva, Switzerland;; 7 Division of General Pediatrics and Adolescent Medicine and Division of Global Pediatrics, Department of Pediatrics, School of Medicine and Public Health, University of Wisconsin-Madison, Madison, WI, USA;; 8 UNITAID, Geneva, Switzerland;; 9 Treatment Action Group, New York, NY, USA.

**Keywords:** tuberculosis, paediatrics, rifamycins, acceptability, prevention, treatment

## Abstract

Historically, there has been considerable delay between stringent regulatory authority approval for new TB drugs in adults, and subsequent trials, registration, and access for children. This has been due to the exclusion of older children from phase 3 trials, the failure to study novel drugs in younger children earlier on, and delays in developing and testing paediatric formulations. Specific barriers include the failure to develop paediatric investigational plans early on, regulatory barriers, inefficient trial design and implementation, limited site capacity, and lack of advocacy and funding. The successful implementation of paediatric clinical trials depend on timely access to appropriate paediatric formulations. We review the delayed evaluation of rifapentine in children and describe barriers, costs in time and resources, and lost opportunities resulting in delayed clinical trials and access in the field, and highlight avoidable delays. Lessons regarding enablers and inhibitors from the rifapentine experience could prevent similar delays in future.

Historically, there has been considerable delay between stringent regulatory authority approval for new TB drugs in adults, and subsequent trials, registration, and access in the field for children. This has been due to several factors, including the inappropriate exclusion of adolescents and older children from phase 3 trials, the failure to specifically study novel drugs in younger children earlier on, delays in developing and testing paediatric formulations, and a lack of funding for paediatric TB research.^1^ For instance, there was a 10-year delay for the use of bedaquiline for treatment of children with multi-drug resistant TB from the time bedaquiline was first licensed for adults in 2012 until it was conditionally recommended by the WHO for children in 2022. Specific barriers to children’s inclusion in TB trials include the failure to develop appropriate paediatric investigational plans for novel drugs early on, perceived or real regulatory barriers, a lack of efficiency in trial design and implementation, limited capacity including sites for paediatric trials, and lack of advocacy and funding. The successful implementation of clinical trials of pharmacokinetics, safety, and efficacy of new drugs or regimens in young children depend on the timely access to appropriate paediatric formulations, which may be developed for evaluation in trials and with the intent to be eventually approved and commercialised. The lack of appropriate child-friendly formulations may significantly delay much-needed trials and thus access to new drugs and regimens in children.

## THE RIFAPENTINE EXPERIENCE IN CHILDREN

We focus on the example of rifapentine and examine reasons for the delayed evaluation of rifapentine-containing regimens in young children. We describe barriers experienced, costs in time and resources, and lost opportunities for paediatric drug development resulting in delayed timelines to clinical evaluation and eventual access to marketed formulations in the field. We highlight specific aspects that could have avoided much of this delay including earlier stage considerations that should have been embedded in paediatric drug development planning, engagements with regulators, and clearer messaging to industry and communities. Lessons learnt regarding enablers and inhibitors from the rifapentine experience could be applied to prevent similar delays in ongoing evaluation of other drugs and of novel anti-TB drugs and regimens in children in future (including pretomanid, BTZ-043, quabodepistat, ganfeberole, delpazolid, and sutezolid).

## INDICATIONS FOR THE USE OF RIFAPENTINE FOR TB

Rifapentine, a potent rifamycin with long half-life, was first discovered in the 1960s and has shown substantial benefit in TB preventive treatment (TPT) and in the treatment of TB disease. TBTC Study 26 tested a 12-week, once-weekly regimen of rifapentine plus isoniazid (3HP), demonstrating non-inferiority, good safety, and higher completion rates of 3HP compared to 9 months of daily isoniazid (9H) to prevent TB among adults and children aged 2–17 years.^2,3^ These findings led the WHO to endorse 3HP as a first-line TPT regimen in 2020.^4^ However, critical knowledge gaps regarding 3HP remained in children below 2 years of age and in children living with HIV (CLWH), that is, those children with the highest risk of developing TB and disseminated disease. The exclusion of young children from Study 26 was largely due to the lack of child-friendly rifapentine formulations at the time. In the trial, the 150 mg solid rifapentine tablet (Priftin, Sanofi) was crushed and dissolved to administer to young children down to the age of 2 years since no child-friendly formulation was available. This process was seen as challenging and led to reduced bio-availability of at least 25% in children,^5^ potentially requiring dose adjustments. This formulation also had a bitter taste, highlighting the need for dispersible, taste-masked formulations with equivalent bio-availability.

Recognising that the lack of data on rifapentine in children under 2 years of age presented a barrier to universal access to 3HP, the Community Research Advisors Group (CRAG), the community advisory board to the TBTC, advocated strongly for the network to undertake a clinical trial establishing the safety and optimal dosing of 3HP in children of all ages and in CLWH. The CRAG highlighted this as a top priority with network leadership and participated actively in protocol development and supported clear messaging during the course of trial implementation.

TBTC Study 35 (NCT03730181), a phase I/II pharmacokinetic, safety, and acceptability study of 3HP in children 0–12 years of age and in CLWH, opened in 2019 and was completed in 2024. TBTC Study 35 provided definitive data on dosing, safety, and acceptability of 3HP, leading to an expansion of dosing guidance provided by the WHO in 2024 for children across the entire age spectrum and in CLWH, using novel WHO-proposed pragmatic weight-banded dosing harmonised across disease areas.^6^ Rifapentine doses of up to 40 mg/kg were required in children <2 years to reach adult pharmacokinetic targets, compared to approximately 20–25 mg/kg in older and heavier children. Substantial rifapentine dose increases were required in children below 2 years of age following interim analysis. In contrast, isoniazid doses were standard at 15–25 mg/kg across all weight bands. Thus, doses required for rifapentine and isoniazid to achieve the targeted adult exposure were dramatically different in young children.

Rifapentine interacts with dolutegravir, recommended for first and second-line treatment of HIV by the WHO. DOLPHIN Kids (NCT05122767) is evaluating this interaction and the need for dose adjustment in children <18 years of age. Due to delays in the availability of a rifapentine formulation and dosing, children <2 years of age were studied in a separate cohort in DOLPHIN Kids. Due to dosing, formulation and funding timelines, once-daily dosing of dolutegravir was not studied in CLWH <2 years of age in the trial.

Rifapentine also forms part of the 1HP regimen, one month of daily rifapentine and isoniazid for TPT. This regimen has been evaluated in adults and adolescents down to 13 years of age with HIV (BRIEF TB, A5279),^7^ and more recently, in a trial of pregnant women living with HIV (DOLPHIN Moms, NCT05122026).^8^ Paediatric evaluation of 1HP in dose-finding and safety studies will open to enrolment in 2026 (IMPAACT 2024, NCT07124559). Rifapentine is also indicated in people 12 years and older for the treatment of TB using a 4-month treatment regimen of daily isoniazid, rifapentine, moxifloxacin, and pyrazinamide (HPMZ) based on findings from TBTC Study 31 (NCT02410772).^9^ Again, children <12 years were excluded, in part based on the lack of child-friendly dispersible formulations, and thus pharmacokinetic and safety data are also lacking for this regimen in children. The HPMZ regimen is currently being studied in SMILE-TB (NCT06253715), a phase II/III seamless trial where children <10 years of age with pulmonary and/or peripheral lymph node disease will receive 8 weeks of this regimen compared with the standard of care, based on observed efficacy in low and medium bacillary mouse models. In summary, the potential use case for rifapentine is broad, with considerable potential market for TPT and treatment, once robust data are available from dosing and safety studies in children.

## ENABLERS AND INHIBITORS FOR APPROPRIATE PAEDIATRIC FORMULATION DEVELOPMENT

Rifapentine formulations used in TBTC Study 35 and in DOLPHIN Kids were developed for the trials by Sanofi, the drug originator, and were anticipated to become commercially available following successful trial completion. The principal formulation developed was a fixed-dose unscored combination of rifapentine and isoniazid at 1:1 ratio. Given the differential dosing requirements for rifapentine and isoniazid in young children, with considerably higher mg/kg required for rifapentine in lower weight bands, the company then also developed a second dispersible unscored 100 mg standalone rifapentine formulation to ‘top up’ the fixed-dose combination (FDC) to ensure adequate rifapentine exposure without risking isoniazid over-exposure in the trial (Figure 1). Nested work in TBTC Study 35 indicated that the mango-flavoured trial formulations were highly palatable and acceptable to children with and without HIV, and also to caregivers and health care workers.^10^

**Figure 1.**
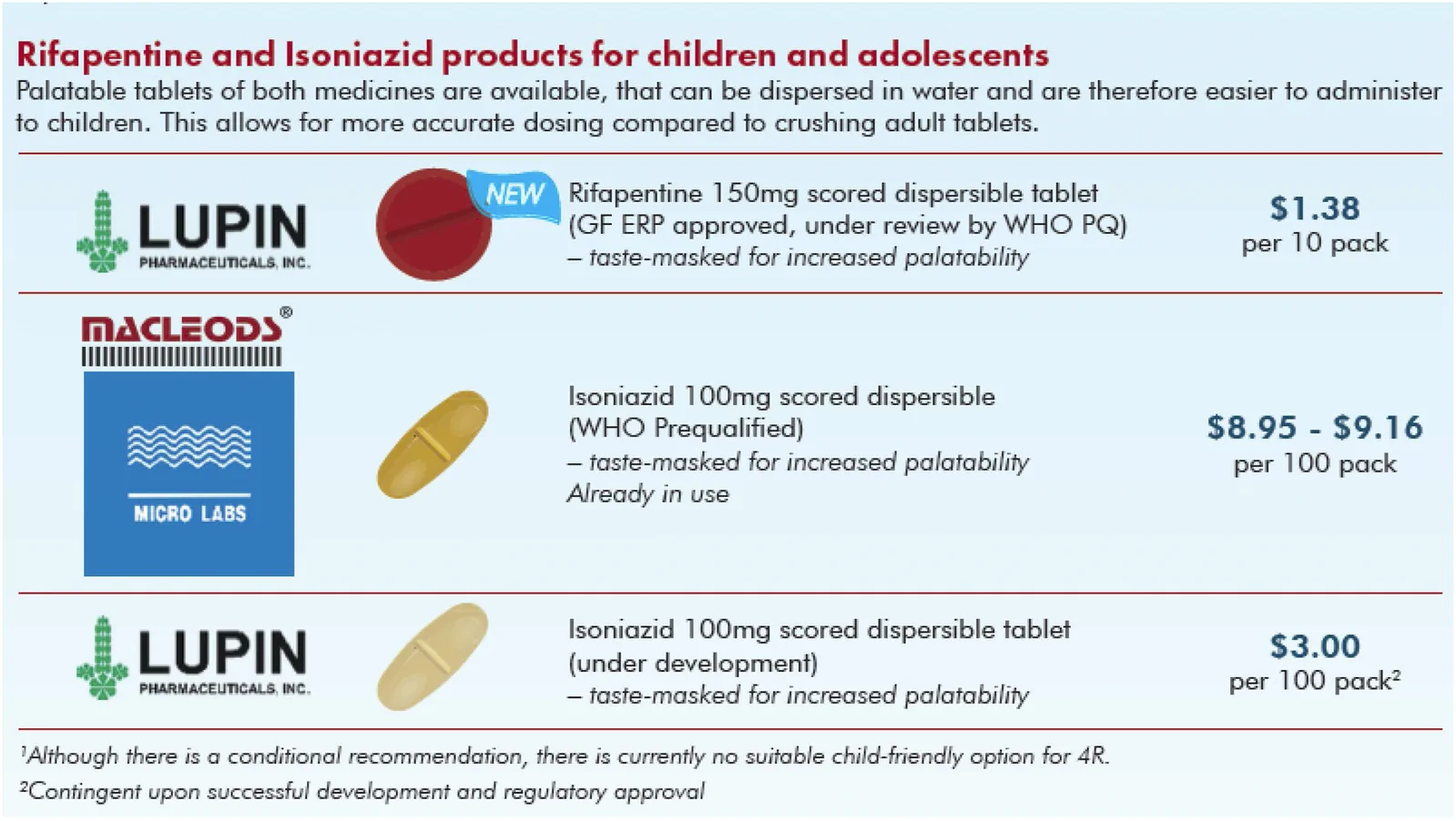
Example of novel generic paediatric rifapentine formulations and costs of rifapentine and isoniazid when procured through the Global Drug Facility at the time of new product introduction. WHO PQ = WHO Prequalification; GF ERP = Global Fund Expert Review Panel.

The complexity of developing a dispersible paediatric formulation depends on the drug’s inherent characteristics. Appropriate excipients should be used, taste masking implemented, and bioequivalence to the reference adult formulation demonstrated (in this case, the solid 150 mg rifapentine tablet – Priftin, Sanofi). Finally, stability data should be available for at least 6 months. This process typically requires a minimum of 2 years. Sanofi invested considerable resources in the paediatric trial formulations. The total development and clinical supply cost was approximately USD 1.5 million for the rifapentine and isoniazid dispersible FDC 150/150 mg tablet and approximately USD 430 000 for the rifapentine standalone dispersible 100 mg tablet. The development timelines for the FDC were from August 2010 (initiation) to August 2019 (first clinical trial supply) and from March 2014 (initiation) to Aug-2019 (first clinical trial supply), respectively, highlighting the considerable delay in paediatric rifapentine formulation development and trial implementation. This formulation development pathway, prioritising the rifapentine FDC, which did not provide appropriate dosing for both drugs across weight bands, resulted in additional investment for the standalone rifapentine formulation. The delays in development of a fit-for-purpose dispersible rifapentine formulation, complexities in obtaining multiple in-country regulatory approvals, the use of modified age-de-escalation instead of no age-de-escalation, the need for multiple interim analyses, and challenges in trial implementation including the COVID-19 pandemic and determination of a nitrosamine impurity, with pauses in enrolment, resulted in a timeline of more than a decade for access to 3HP in young children.

## ADDITIONAL BARRIERS EXPERIENCED AND SOLUTIONS IN TRIAL IMPLEMENTATION AND COMMERCIAL PRODUCTS

The rifapentine formulation development and clinical trial implementation pathway was also impacted by the emerging issue of nitrosamine impurities. This also remains an ongoing issue for commercial products. Nitrosamine impurities, which are potential carcinogens, were first identified in pharmaceutical products in 2018. Regulatory authorities and WHO Prequalification (PQ) have issued guidance documents on the identification and mitigation of nitrosamine impurities in drug products, including risk assessment, confirmatory testing, and controls to reduce the nitrosamine impurity levels in drug substances and drug products. Currently, WHO PQ allows an interim limit of 1-cyclopentyl-4-nitrosopiperazine, the nitrosamine impurity identified in rifapentine products, of not more than 20 ppm. The WHO PQ requires that the same interim limit applies to children as to adults.^11^ The identification of a nitrosamine impurity led to additional safety reviews, protocol amendments, and delays in trial implementation to allow for updated information in informed consent documents and engagement with community stakeholders. Succinct information documents and tools were developed by TAG, WHO, Cling Health Access Initiative (CHAI), and others, to provide transparent and accessible information to stakeholders. Input was provided from community advisory groups on appropriate practical language changes in informed consent documents.

## TRIAL COMPLETION AND GLOBAL RECOMMENDATIONS DO NOT EQUATE ACCESS IN THE FIELD

The successful completion of rigorous pharmacokinetic, safety, and acceptability studies of new drugs or regimens in children which result in updated global guidelines is however not sufficient to guarantee access to commercially available quality-assured (i.e., using internationally recognised standards) formulations in-country. The development of anti-TB drug formulations by industry is driven by several factors, including global and in-country guidelines, listing on WHO and national essential medicines lists (the latter typically depending on in-country approvals), perceived global market share, incentives (direct or indirect), regulatory requirements for approval, patents, and the actual cost of developing formulations. Guaranteed procurement mechanisms, for example, through the Global Drug Facility (GDF), are useful to ensure centralised procurement, to ensure sustained markets and to ensure reasonable cost once a new formulation is WHO Prequalified, as a minimum.

Ultimately, the rifapentine paediatric formulations used in TBTC Study 35 and Dolphin Kids were not submitted for WHO PQ and regulatory approvals, or marketed commercially. The primary patent on Priftin was filed in 1976 and expired 20 years later. However, Sanofi remained the sole supplier of quality-assured rifapentine and, in 2014, filed two patent applications on FDCs of rifapentine and isoniazid, one formulated for adults and the other for children, in 69 countries. Following a global advocacy campaign and a series of patent oppositions in India, Thailand, and several other countries, Sanofi announced in 2020 that it was voluntarily withdrawing its 3HP FDC patent applications everywhere where they remained pending and would abandon patents in countries where they had already been granted.^12,13^ This paved the way for generic production and greater market competition by removing any lingering ambiguity concerning freedom-to-operate.

## LESSONS LEARNT TO ENSURE EARLIER ENGAGEMENT WITH MANUFACTURERS AND IMPROVE CLARITY REGARDING PRIORITY FORMULATIONS NEEDED

In 2021, the WHO-convened Paediatric Drug Optimization for TB (PADO-TB) group – which includes clinical researchers, market-shaping entities, regulators, and funders, among others – discussed and aligned on the priority formulation of rifapentine to be encouraged for generic development. The group agreed that a 150 mg functionally scored, dispersible rifapentine standalone tablet should be prioritised for generic development. This formulation would provide dosing flexibility across multiple indications for TB prevention and treatment in children across all ages.^14^ Although FDCs can improve supply chain efficiency and support adherence, a rifapentine-containing FDC was not listed as a priority formulation for development in the short term. This was because, given different dosing ratios expected for rifapentine-containing TPT regimens (3HP, 1HP) and different dosing ratios expected for younger and older children who are given the same regimen, there would be a potential need to develop another rifapentine-including FDC, if a regimen composed of HPMZ would be recommended by the WHO for children with TB in future, posing the risk of disincentivising manufacturers and fragmenting markets. Following the PADO-TB meeting in 2021, the WHO updated the list of products eligible for WHO PQ (WHO PQ expression of interest) and clearly signalled to manufacturers the need to develop such formulations.

Product development, manufacturing capacity, commercialisation, and market shaping were ultimately essential to bring paediatric rifapentine formulations to market. These, along with the paediatric trials, should have been undertaken earlier. Through the IMPAACT4TB project, funded by Unitaid, a collaborative partnership was initiated between CHAI and Lupin Limited to accelerate the development, WHO PQ filing, and commercialisation of a child-friendly, paediatric rifapentine 150 mg scored, dispersible tablet. CHAI provided technical and regulatory support for the paediatric rifapentine product while Lupin transparently shared the product data, reports, and regulatory dossier for review prior to WHO PQ filing. Lupin submitted the paediatric rifapentine product to the WHO PQ and the Global Fund Expert Review Panel (GF ERP) in June 2023. The GF ERP approval was obtained in November 2023, allowing time-limited procurement while the WHO PQ review was in progress. Lupin commenced commercial production following GF ERP approval. WHO PQ was obtained in November 2024. National regulatory filings were conducted in parallel and are ongoing in several high-burden countries. Lupin’s paediatric rifapentine 150 mg dispersible tablet is functionally scored which allows for dose increments of 75 mg. It disperses quickly in small amounts of water and has a raspberry-mint flavour. IMPAACT4TB also developed education and training materials including product briefs, clinician and patient guides, and a dosing wheel to support field implementation. A second formulation (strawberry-flavoured) was developed by Macleods Pharmaceuticals, India, and obtained WHO PQ in July 2025.

## PERSON-CENTRED APPROACHES TO INVOLVEMENT OF STAKEHOLDERS IN FORMULATION DEVELOPMENT

PADO-TB also highlighted the need to account for acceptability, including palatability, of new paediatric formulations. The new paediatric rifapentine formulations disperse rapidly in small amounts of water and are both taste-masked for bitterness. Inclusion of patient-centred outcomes in clinical trials, including acceptability of formulations, is important. TBTC Study 35 included important data on the acceptability of the trial formulations among children, their caregivers, and health care workers, also in CLWH, indicating excellent palatability and overall acceptability of the mango-flavoured Sanofi rifapentine formulations.^10^ However, the lack of scoring of tablets provided practical challenges to dosing children (Figure 1). Early work on acceptability and palatability in children is needed at the time paediatric formulations start development, to inform optimal taste masking, rather than after formulation development has been completed. Such work could include ‘swish and spit’ studies of multiple formulation options in children, who can actively participate in formulation preference clinical observational studies to inform formulation taste selection even at a young age.^15^ Earlier and more effective community engagement is critical to ensure that children are appropriately included earlier in TB therapeutics research and that appropriate formulations are developed early on. The role of advocacy groups including the TBTC CRAG, local community advisory boards where trials were conducted, and other community representatives through the IMPAACT4TB project and Unitaid was important. Such efforts should be expanded and be more inclusive of affected communities, children, and their families going forward in paediatric therapeutics research.

## SOLUTIONS TO SECURE INVESTMENT FOR MARKETING AND ACCESS

The IMPAACT4TB collaborative partnership accelerated the development, WHO PQ filing, and commercialisation of the child-friendly, paediatric rifapentine 150 mg scored, dispersible tablet. To secure access pricing and accelerated uptake, guaranteed volumes of paediatric rifapentine were donated through a GDF-run early market access vehicle. Current prices for the new adult and paediatric rifapentine formulations were negotiated through the GDF, where large volumes of quality-assured drugs can be guaranteed for suppliers and countries through a central procurement platform with rigorous WHO PQ mechanisms (Figure 2). If countries do not procure through the GDF and use in-country tender processes, prices may vary and be higher and there may be a lack of continuity in supply.

**Figure 2.**
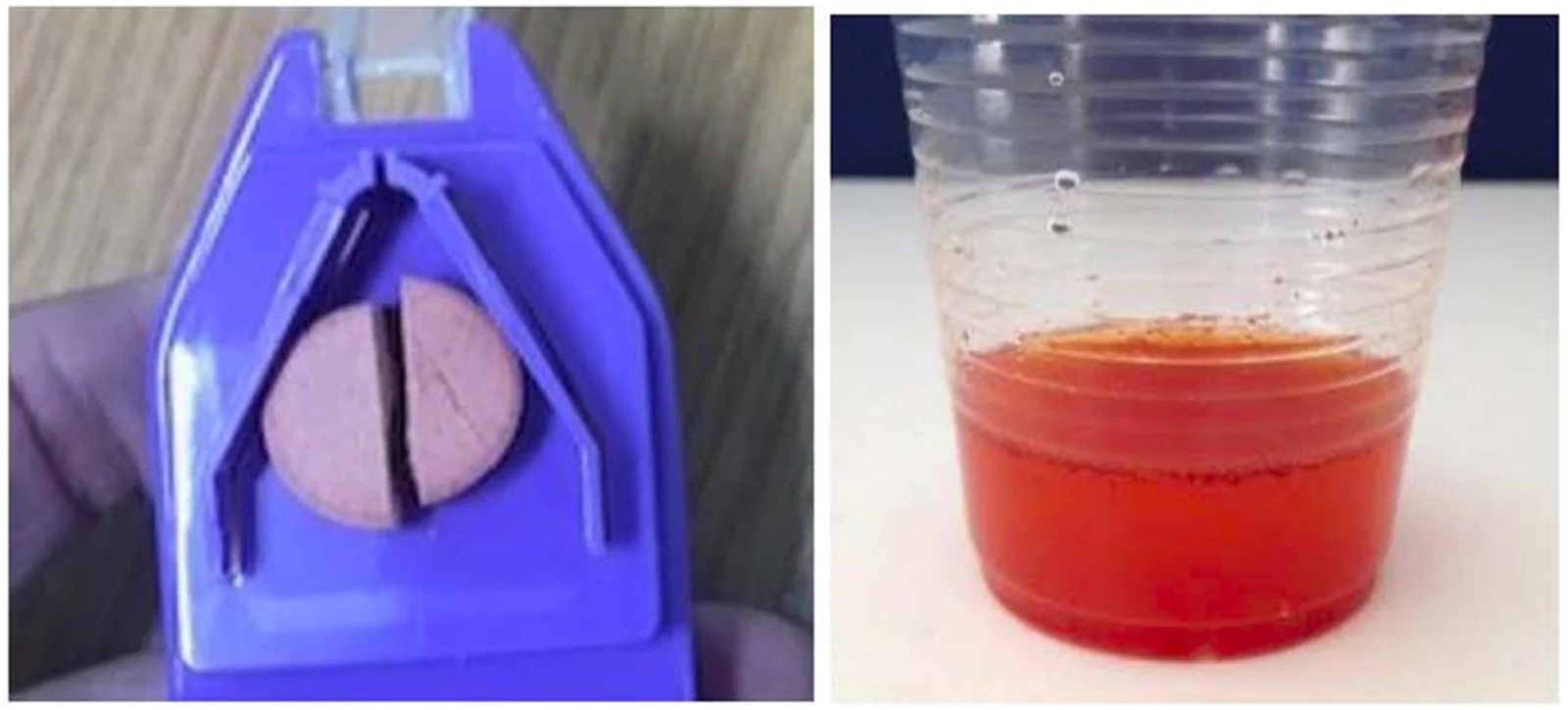
Paediatric rifapentine dispersible mango-flavoured 100 mg formulation used for the TBTC Study 35 trial. The formulation is unscored and required splitting, demonstrated using a pill cutter (left) and dispersed in water (right).

## REGULATORY BARRIERS AND GLOBAL ACCESS NEEDS FOR CHILDREN

TBTC Study 35 was conducted under a United States Food and Drug Administration Investigational New Drug application. Rifapentine does not currently have approval from the European Medicines Agency. The current US FDA-approved label for the Sanofi product includes 3HP dosing only for children 2 years and older. Thus, the lack of regulatory submission and approval in Europe and in low- and middle-income countries (LMICs) and also in the USA for children older than 2 years has resulted in lack of access to 3HP in children globally, not just in LMICs. In many LMICs, medicines are only prioritised for essential medicines lists and procurement if they have first been registered and approved by national regulatory authorities, in addition to the WHO PQ process. The cost for submission of a dossier for registration could be seen as prohibitive for generic manufacturers in the absence of additional investments given the large number of potential countries who require these processes and the perceived small volumes for the paediatric market following market authorisation by manufacturers.

## LESSONS LEARNT AND THE WAY FORWARD TO REDUCE DELAYS IN PAEDIATRIC ACCESS

There was a delay of 11 years following the completion of TBTC Study 26 conducted in adults and in older children, to obtaining dosing and safety data in young children and CLWH, and having commercially approved rifapentine formulations. Timelines for access to 1HP and HPMZ regimens are also still unacceptably long (Table 1). More rapid development of a suitable paediatric rifapentine formulation at the time the phase 3 trial opened would have dramatically accelerated the clinical development programme in children. In addition, the development of a standalone rifapentine formulation only by industry would have avoided unnecessary costs and further delays in conducting the clinical trials.

**Table 1.** Timelines for stringent regulatory approval to paediatric access for rifapentine in children across treatment indications.

	Rifapentine in 3HP (prevention)	Rifapentine in 1HP (prevention)	Rifapentine in HPMZ**^A^** (17-week treatment): severe and non-severe TB
Stringent regulatory authority or WHO adult indication/approval	2014	2019	2022
Paediatric study open	2019	2026	2027
Paediatric study results	2024	2028 (estimated)	2029 (estimated)
Time from adult indication until children of all ages can benefit	11 years	9 years (estimated)	7 years (estimated)

3HP = 3 months of once-weekly isoniazid plus rifapentine; 1HP = 1 month of isoniazid plus rifapentine; HPMZ = isoniazid, rifapentine, moxifloxacin, and pyrazinamide.

A HPMZ is also currently being evaluated as an 8-week regimen in children in the SMILE-TB trial (NCT06253715).

Better alignment among stakeholders and clearer messaging and advocacy regarding the need for investment in priority paediatric formulations – in this case, a functionally scored standalone rifapentine tablet – would have reduced the overall timeline for access in young children. The WHO-led PADO-TB process provided clear messaging to generic manufacturers regarding global needs and priority formulations. A systematic application of this process moving forward will allow for clear signalling for products that are under investigation in adults. In addition, more rapid targeted licensing agreements and the determination of the potential market size early on – TPT for drug-susceptible TB as part of 3HP (and eventually also for 1HP and treatment of disease as part of the HPMZ regimen) – may have incentivised generic companies to pursue formulation development earlier on. The market for TPT in children is substantial. The WHO estimated that 1.7 million children below 5 years of age needed TPT in 2024; of these, 5, 652,000 (38%) were initiated on TPT.^16^

There is clear international agreement that ‘all medicinal products should be designed to meet patients’ needs and the intended product performance’.^17^ Paediatric trials of new anti-TB drugs typically cannot begin in young children without appropriate formulations. Critical decisions must be made related to the type of product, dose strength of product/s, and taste masking requirements based on advice given by consensus processes like PADO-TB and where needed, ad hoc target product profiles. Uncertainty about preferred characteristics results in delays, and addressing these issues prior to development may require costly and time-consuming processes such as developing new paediatric formulations and submission to regulatory authorities. This ultimately wastes already limited resources in a global health funding landscape facing severe challenges, and delays access to innovations for children affected by TB. Other sources of delay should also be addressed to expedite access in children.

Designing and implementing efficient and optimally designed pharmacokinetic, safety, and acceptability studies in young children earlier on should be prioritised and expedited. For new anti-TB drugs, efficacy can be extrapolated from adults and older children, and efficient dosing and safety studies in children should be conducted early and in a coordinated manner without the need for repeat efficacy studies, and with the appropriate formulations.^18^ The historical use of age de-escalation should be replaced by simultaneous enrolment of age groups according to weight bands since doses in older children do not inform doses in young children and pharmacokinetics and side effects are unlikely to substantially differ above 2 years of age. Conducting clinical trials of novel anti-TB medicines in children is a global priority. However, this is necessary but not sufficient to ensure access to much-needed treatments in the field. Parallel investments should be made to develop and expedite the introduction and roll-out of commercially approved and affordable paediatric formulations to ensure access and global impact (Table 2).

**Table 2.** Key lessons learnt in the evaluation of rifapentine in children.

Paediatric rifapentine formulations should have been developed at the time phase 3 trial was implemented
Children and adolescents ≥10 years should be included in phase 3 TB trials
Young children should be included early on in pharmacokinetic/safety studies
Trials should be enriched for children <2 years of age, given greatest biological variability
Age-de-escalation design should be avoided and all age groups enrolled in parallel
Development of fixed-dose combination formulations is problematic if they do not support accurate dosing of multiple drugs across paediatric weight bands
Development of a standalone dispersible and functionally scored rifapentine tablet should have been a priority.
Including acceptability and palatability work early on to inform formulation development is important
Completion of clinical trials in children and global guideline changes do not automatically result in access to these drugs
Early effective stakeholder engagement is critical to ensure that children are appropriately included in clinical trials
Central procurement mechanisms such as the Global Drug Facility can ensure reasonable prices and continuity of supply of quality-assured anti-TB drugs

## CONCLUSION

Coordination between researchers, funders, industry, normative, regulatory, procurement, advocacy groups, and civil society is important. Key enabling mechanisms, flexible research platforms, and working groups that enable the rapid translation from TB clinical trial evidence to access will save time, money, and children’s lives.
